# Name matters! The cost of having a foreign-sounding name in the Swedish private housing market

**DOI:** 10.1371/journal.pone.0268840

**Published:** 2022-06-08

**Authors:** Hemrin Molla, Caroline Rhawi, Elina Lampi

**Affiliations:** Department of Economics, University of Gothenburg, Gothenburg, Sweden; Ghent University: Universiteit Gent, BELGIUM

## Abstract

Both immigration and a troubling housing deficit have increased rapidly in Sweden over the past 20 years. In this internet-based field experiment, we investigated whether there exists discrimination in the Swedish private rental housing market based on the names of apartment seekers. We used a correspondent test by randomly submitting equivalent applications from four fictitious, highly educated, and seemingly “well-behaved” male applicants in response to a number of randomly selected private housing ads. Each advertising landlord received applications from two applicants with names signaling Swedish, Arab/Muslim, Eastern European, or East Asian ethnicity. Our results show that the person with a name associated with the dominant ethnic group received most callbacks from the landlords, while the persons with Eastern European- and East Asian sounding names, and especially the Arab/Muslim-sounding name, yielded significantly lower callback rates. Moreover, each applicant’s callback rates are about the same regardless of whom he was paired with, reinforcing our result that a person’s name clearly matters when applying for an apartment. The comparisons with previous discrimination research focusing on the Swedish housing market show that the situation for a male person with an Arabic/Muslim-sounding name has at least not improved in Sweden in the past decade.

## 1. Introduction

The housing market is one of the many domains where ethnic discrimination has been proven to occur. This can have some serious consequences, as it limits the opportunities for some people for example in the labor market. About one fifth of the Swedish population 15–34 years old have at some point decided not to apply for a job due to the housing situation in the biggest Swedish cities [1]. Thus, when individuals with an ethnic minority-sounding name find even greater difficulties than ethnic Swedes when trying to rent an apartment in cities with a large number of employers, it could result in a more segregated labor market. A field experiment study on discrimination investigated callback rates for several immigrant groups in the French housing market [2]. In addition to a person with a traditionally French name, they used both male and female applicants with names signaling affiliation with five Southern European main immigrant groups in France, namely Northern and Sub-Saharan African, Middle Eastern (Turkish), Eastern European (Polish), and Southern European (Portuguese and Spanish) people. Each application email included only the name of the applicant. However, they wrote in the email that an applicant would only be available for contact during evenings, which could be taken to indicate that the applicant was employed. The researchers found significant variation in callback rates across the immigrant groups and that some groups clearly had higher search costs, especially individuals with Northern African-, Sub-Saharan African-, or Turkish-sounding names.

In addition, a large literature shows that especially apartment seekers with Arab/Muslim-sounding names tend to receive fewer callbacks from landlords compared with the dominant ethnic group in a country. Meta-analyses of 25 studies about discrimination in rental housing markets in the OECD countries found that individuals belonging to the ethnic majority were more than twice as likely to be favored than applicants with Arab/Muslim-sounding names with identical characteristics [3]. All these studies used field experiments involving written application letters (correspondence studies). The other way to test for possible discrimination is to use a personal approach (audit studies) where test persons who are identical in as many characteristics as possible except ethnicity apply for the same rental apartments. For an overview of previous studies on ethnic discrimination in the housing market that have utilized a personal approach, see [4]. However, the personal approach has been criticized due to the possibility that the test persons still differ in several unobservable characteristics and because it is impossible to ensure that the test persons communicate with the landlords in identical ways [4–7]). Due to this criticism and since the internet is currently the main channel for marketing all kinds of housing in Sweden, we choose to use a correspondence study approach in this paper.

Previous studies have also found that this type of discrimination did not decrease when a person with an Arab/Muslim-sounding name had a high-status education and/or job [8], or that the discrimination gap between ethnic majorities and minorities decreased with a high-status education and/or job, but only partly [9–13]. Thus, although many previous studies have shown that individuals with a high degree of education enjoy greater opportunities for housing, there remains a discrimination gap between individuals with an ethnic majority-sounding name and those with an ethnic minority-sounding name when they have the same degree of education. If individuals with an ethnic minority-sounding name feel forced to turn down (high-yield) job offers due to difficulties finding housing, it tends to generate an even more segregated society, which in turn increases the social gap.

In the labor market literature, there are some studies investigating possible discrimination among multiple ethnic minority groups. For example, a study investigated discrimination in the Australian labor market among four minority groups in addition to the native majority group and found that members from all the minority groups needed to apply for significantly more jobs than the ethnic majority. Furthermore, the level of discrimination varied across the minority groups [14]. Most field experiments concerning discrimination have included only one or two ethnic minority groups [15]. In contrast, Thijssen, Coenders, and Lancee [15] investigated ethnic discrimination among 35 ethnic groups in the Dutch labor market. They found that applicants of native-majority origin had by far the greatest chance of being contacted by potential employers in response to a job application. Despite identical qualifications, Western minorities received significantly fewer responses, and those of non-Western origin fared even worse. Another recent paper, in sociology, found similar pattern in the Finnish labor market among five different ethnicities [16].

Sweden has adopted international conventions and stipulated laws to prevent discrimination. However, studies conducted in Sweden show results similar to those found elsewhere in the world: persons with names associated with an ethnic minority have greater difficulties finding an apartment than others. Previous research on discrimination in the Swedish housing market has mainly focused on ethnic discrimination related to differences in responses between apartment seekers with Swedish-sounding and Arab/Muslim-sounding names. These studies have identified a clear discrimination of people with Arab/Muslim-sounding names compared with people with Swedish-sounding names [7, 8, 17, 18].

We use an internet-based field experiment to investigate whether there exists discrimination in the Swedish private rental housing market, and if so, whether there are differences based on the names of apartment seekers. We do this in regions of the three largest cities in Sweden.

This research contributes to the literature in two ways. First, with the exception of few papers [2, 19], we identify a lack of research where several ethnic minorities are investigated in housing markets in general and especially in Sweden. This is a shortcoming since the population in many European countries is becoming increasingly ethnically diverse and it is not clear yet whether discrimination affects all ethnic minority groups equally [15]. Thus, in this study we investigate whether only people with Swedish-sounding names and those with Arab/Muslim-sounding names are treated differently in the rental market or if similar differences can be found when comparing other pairs of ethnic groups. To this end, in addition to the Arab/Muslim-sounding name, we used one Eastern European-sounding and one East Asian-sounding male name.

Secondly, Sweden has become an interesting object for studies of potential ethnic discrimination. Historically, the country has experienced substantial emigration and a low rate of immigration. In the last few decades, however, Sweden has rapidly changed from being a country with a rather homogenous ethnic population to being a country with up to 33 percent immigrants in the largest cities. Nationwide at the end of 2018, 19.1 percent of the Swedish population had been born outside of Sweden [20].

In 2007–2008, when the previous studies of discrimination of people with Arab/Muslim-sounding names in the Swedish housing market were conducted [7, 8], Sweden received about 100,000 immigrants per year. An additional study [18] was conducted in 2010 and 2011, and at that time the numbers were below 100,000. The corresponding numbers in 2016 and 2017, i.e., the years immediately before our study was carried out, were higher than ever before or after: 163,000 and 144,500, respectively [21].

The historically large immigration in the past 10 years together with too low rates of housing construction has increased the demand for rental housing units dramatically. In addition, another change compared with the previous studies on discrimination in the Swedish housing market is that over the course of the three latest general elections in Sweden, national conservative right-wing party the Sweden Democrats has become the country’s third largest party. In 2010, the Sweden Democrats gained their first seats in the national parliament after winning 5.70 percent of the Swedish people’s votes, and in the 2018 general election they won 17.53 percent of the votes [22, 23]. However, a study conducted in Belgiumdid not find more ethnic discrimination in municipalities with more negative attitudes towards Belgian immigrants [24].

It is possible that the previously identified discrimination in the Swedish rental housing market has either decreased or increased in recent years, or that the discrimination has moved from one ethnic minority group to another. In addition to investigating callback rates for several ethnic minority groups in the Swedish housing market, the second aim of this study is to investigate, by comparing our results with those in Ahmed, Andersson, and Hammarstedt [8], whether we can confirm the previous findings about discrimination in the housing market based on applicant name or the opportunities in the housing rental market have improved in Sweden in the past 10 years for a male applicant with an Arab/Muslim-sounding name.

The experiment was conducted by using three different but equivalent application emails that were randomly sent to 620 private landlords. Further, we used four different fictitious male persons with names signaling different ethnicities as applicants for vacant rental units advertised on the popular Swedish buy-and-sell website Blocket.se. Ethnicity was solely signaled through the name of the applicants and not by providing for example country of birth. Thus, our fictitious persons could have been born either in Sweden or elsewhere. Each landlord received applications from two of our four fictitious persons, which enables us to test whether the likelihood of receiving a callback from a private landlord varies depending on the ethnicity associated with the name of applicants. Each pair of fictitious persons was randomly matched with a landlord.

Our results clearly show that there is heterogeneity in discrimination in the Swedish housing market across the different immigrant groups. The Eastern European- and the East Asian-sounding names, and especially the Arab/Muslim-sounding name, yielded significantly lower callback rates than the name signaling membership of the dominant ethnic group, i.e., ethnic Swedes. Secondly, the comparisons with the Ahmed, Andersson, and Hammarstedt [8] paper shows that the situation for a male person with an Arabic/Muslim-sounding name has not improved in Sweden over the past decade.

The rest of this paper is organized as follows: In Section 2, we describe our experimental design and how the data was collected. Section 3 is the results section and Section 4 concludes the paper with a discussion.

## 2. Method, experimental design, and data collection

### 2.1. Method and emailing procedure

This study used the correspondent test by randomly sending out equivalent applications from four fictitious applicants to landlords who had posted housing ads on the popular Swedish buy-and-sell website Blocket.se. All the fictitious persons used were presented as having non-problematic backgrounds and good jobs and as being “well-behaved” and highly educated, in order to eliminate negative responses from the landlords based on factors unrelated to ethnicity. All the landlords were private landlords advertising either a rental apartment or rental of a condominium house/part of a house they owned. We submitted 1,240 applications in response to 620 private housing ads posted November 8–November 15, 2017. Each landlord received two different applications from two different male applicants with names signaling different ethnicities. One of the persons had a Swedish-sounding name (Johan Andersson), one had an Arab/Muslim-sounding name (Ali Hassan), and the remaining two an Eastern European-sounding name (Milan Mladenovic) and an East Asian-sounding name (Yong Wang).

While there might be a risk of making a landlord suspicious by sending two applications to the same landlord, varying the ethnic makeup of applicant pairs enables us to better measure whether the degree of discrimination a certain applicant faces changes depending on who else responded to the same ad at the same time. Moreover, by sending two emails to the same landlord, we increase the precision in our estimation since, within each applicant pair, unobserved landlord/apartment characteristics are identical for each pair. If everything except the name in the applications is of equal quality, it is possible to conclude that any discrimination found is solely due to the names provided (see, e.g., [8–13, 17]. However, since all applicants were presented as having non-problematic backgrounds and good jobs and as being “well-behaved” and highly educated, our possible findings of discrimination could be underestimations. In addition, the use of applicants who are too similar in relevant aspects (e.g., education or credit history) has been criticized by some researchers. To be able to achieve an unbiased estimate of discrimination, a correspondence study should include observable measures of variation in the quality of applicants [25].

The applications were combined in all possible ways ethnicity-wise for a total of six different pairs. Moreover, in order to avoid discrimination based on general disapproval of any of the stated employers, or of one of the application letters for other non-ethnicity-related reasons, we used three different application letters and the letters and the names where combined randomly prior to each submission of application pairs. Since each landlord received two applications, we also randomized which fictitious person submitted his application first, on average about 4 hours before we sent the application for the other fictitious applicant in the pair. Importantly, since we sent two applications to each landlord, we will test for a possible order effect in our regressions.

The experiment was conducted in the regions of Stockholm, Gothenburg, and Skåne, where the Stockholm region has 2.4 million inhabitants and consists of 26 municipalities, the Gothenburg region has 1.7 million inhabitants and 13 municipalities, and the Skåne region has 1.3 million inhabitants and 33 municipalities. In each region, we find the country’s three largest Swedish cities Stockholm, Gothenburg, and Malmö respectively. In 2018, about 25 percent of all foreign-born individuals in Sweden lived in the Stockholm region, about 25 percent in Skåne’s Malmö region, and about 21 percent in the Gothenburg region [26]. One third of all people living in Malmö, which is the biggest city in the Skåne region and the third largest in Sweden, have immigrant background [27]. The geographic locations of the three regions differ substantially: the region of Stockholm is located on the east coast, the region of Gothenburg on the west coast, and the region of Skåne in south Sweden.

All ads with a stated rent of up to 10,000 SEK/month (€ 1,015/month at the time of the study) and a size of at least 20 square meters were responded to. The only exception was ads where a landlord clearly stated they would only accept female tenants. The name of the city where each applicant had received a new job (Stockholm, Gothenburg, or Malmö) was matched with the location of the vacant rental unit in each letter.

The design of a correspondence study like ours requires that landlords are not informed about the study. However, we do not collect or use any information about the landlords themselves in our study, except about whether the name of a landlord sounded Swedish or not. If the applicant received an answer of any kind, to decrease the inconvenience of the landlord, we immediately sent an email back thanking the landlord and saying that housing had already been found elsewhere. Our project does not fall under the criteria given in the law regarding ethical vetting in Sweden [28].

### 2.2. Choice of names

Four fictitious applicants were created: Johan Andersson, which signals a Swedish background, Ali Hassan, which signals an Arab-Muslim background, Milan Mladenovic, which signals an Eastern European background, and Yong Wang, which signals an East Asian background. We chose these names because it is important to use first- and last names that clearly signal the chosen ethnicity [29] and because they are strongly associated with some of the main ethnic groups currently present in Sweden. The largest immigrant groups (excluding people from the other Nordic countries) are immigrants from Asia (37 percent) and EU countries (32 percent), followed by immigrants from Afghanistan, Iran, Iraq, and Syria (23 percent) [27]. Only about 9 percent of all immigrants living in Sweden today originate from Eastern European countries outside the EU. The backgrounds of our fictitious persons are only signaled via their names, which do not necessarily say anything about country of birth.

The names of the Swedish and Arab/Muslim applicants were chosen from Statistics Sweden. Every year, Statistics Sweden presents statistics on the 100 most common first and last names in the country. Using the most recently (2016) presented list of names, we chose the most common Swedish and Arab names in Sweden: Johan Andersson and Ali Hassan. The names of the fictitious Eastern European and East Asian applicants were chosen through research on the internet, where multiple lists of the most common names of Serbian and Chinese origin were found. From those lists, the most frequently occurring names were chosen after ensuring in Statistics Sweden´s database that at least several hundred persons had these names in Sweden. Of course, there is never a perfect correlation between a name and ethnicity since some names can exist in several ethnicities [29, 30]. In addition, people might perceive names differently when it comes to ethnicity [30].

### 2.3. The fictitious applicants

In order to keep the applicants from being screened out among the large number of housing seekers on Blocket.se, we wanted them to signal desirable characteristics such as a high level of education, a steady income, and a good credit history. In accordance with the method, it was also important that the applicants were made as equivalent as possible. They therefore had the same age, gender, education, marital status, and leisure interests, and similar types of jobs with equivalent wages. All of them were also non-smokers and “well-behaved,” and they had no children or pets. As these characteristics were kept constant in the applications, we believe that any significant differences in response rates found must depend on discrimination, as only the names distinguished the applicants. The age of our applicants (27 years) was motivated by the fact that a 27-year-old person is old enough to have both university education and some work experience. Moreover, the 20–29 year 20–29 year olds make up the largest subtenant age group [31].

Since each advertiser received two applications from two different persons, we could not use identical employers. Instead, similar types of jobs at well-known and established companies were chosen, and all of the jobs could be expected to pay good and similar wages. Our reason for using three different letters was that we wanted to alter between, e.g., three different employers, decreasing the risk that other information than the name of an applicant might influence a landlord. Furthermore, all applicants were searching for housing because of a new job in a new city. In other words, all applications contained the same type of information, but the wording of the emails differed to avoid suspicion. All the applicants also had different email accounts. The three applications used in the experiment can be found in S1 Appendix. Please note that all applications were written in Swedish and have subsequently been translated to English only for the purposes of this paper. For comparison, in S1 Appendix, we also show the application letter used in the Ahmed, Andersson, and Hammarstedt study [8].

## 3. Results

### 3.1. Descriptive results for the whole sample

In total 31 percent of the applications yielded a callback from a landlord. We define a *callback* as a case where a landlord offered an applicant to rent the accommodation, showed an interest in renting out but had a few more questions to ask, or invited an applicant for a showing. Table 1 below reports the number of applications per applicant as well as the callback rates.

**Table 1:** Responses per applicant.

Name	Johan	Ali	Milan	Yong
No. of applications/person	282	298	287	305
Callback rate	39%	23%	31%	31%
P-values for call-back rates				
Johan-Ali	0.000			
Johan-Milan	0.037			
Johan-Yong	0.046			
Ali-Milan	0.041			
Ali-Yong	0.027			
Milan-Yong	0.898			

The table shows that the callback rates were 39 percent for Johan, 23 percent for Ali, 31 percent for Milan and Yong. Results from the two-sample Z-test of proportions show that Johan received significantly more callbacks than any other applicant, while Ali’s callback rate was significantly lower than anybody else’s. There is no significant difference between Milan’s and Yong’s callback rates. We conclude that Johan seems to be most popular among the landlords, followed by Milan and Yong. Ali clearly had the lowest rate of positive callbacks, and the difference is the largest when compared with Johan: 16 percentage points.

Table 2 shows descriptive statistics of the sample. Most of the vacant rental units were located in the Stockholm region, around the capital of Sweden, and more often in a suburb than in a city center. It seems to be more common to rent out a whole apartment than just a room. A majority of the landlords had Swedish-sounding names and the rental units had been advertised online for an average of 12 days before we sent out the application letters. The average number of days an advertisement had been posted before we submitted an application was about the same for each applicant, i.e., 11 days for Milan, 12 days for Johan and Yong, and 13 days for Ali.

**Table 2:** Descriptive statistics of the sample.

Variable name	Variable description	Mean value	Standard deviation
City center	=1 if the rental unit is located in a city center	0.273	
Suburb close city center	=1 if the rental unit is located in a suburb close to the city center	0.323	
Suburb	=1 if the rental unit is located in a suburb further from the city center or in a municipality further way from the big city	0.398	
Stockholm	=1 if the rental unit is located in the Stockholm region, i.e., the capital of Sweden	0.480	
Gothenburg	=1 if the rental unit is located in the Gothenburg region	0.179	
Skåne	=1 if the rental unit is located in the Skåne region	0.341	
Room	=1 if the rental unit is a room only	0.316	
Apartment	=1 if the rental unit is an entire apartment	0.684	
Rental property	=1 if the rental unit is a rental apartment or part of a rental apartment (i.e., the rental ad concerns a subletting arrangement)	0.560	
Condominium	=1 if the rental unit is a condominium or part of a condominium/house (i.e., the rental unit is landlord owned)	0.440	
Rent	Rent for the apartment or room in SEK 1,000	7.198	2.038
Swedish landlord	=1 if landlord had a Swedish-sounding name	0.604	
Landlord unknown ethnicity	=1 if impossible to judge whether a landlord had a Swedish-sounding name	0.142	
No. of days	Number of days a rental unit had been advertised online before the application was sent out.	12.386	13.063
Share of foreign-born	Share of foreign-born citizens in the municipality where the rental unit was located.*	26.567	7.630
Sent application first	=1 if an applicant sent his application first in a pair combination	0.488	
No. of observations	1,172		

* = Source: [32]

### 3.2. Regression results

We will now have a closer look at our data and investigate whether differences in the characteristics of the rental units could explain the likelihood of receiving a callback. We use a binary probit model where the dependent variable is 1 if an applicant received a callback. Since each landlord received two applications, the standard errors in all the regressions are clustered at advertisement (landlord) level. In Model 1, we include only the applicants and Ali is the reference category. In Model 2, we add controls inclusive a control for the order in which applications were sent to a landlord. Marginal effects of both models are shown in Table 3.

**Table 3:** Robust marginal effects from a binary probit regression. The dependent variable is *callback*. Standard deviations in parentheses and they are clustered at advertisement level.

Variable	Model 1	Model 2
Johan#	0.158***	0.153***
	(0.035)	(0.034)
Milan#	0.079**	0.074**
	(0.035)	(0.034)
Yong#	0.084**	0.078**
	(0.034)	(0.033)
Inner city		-0.090*
		(0.049)
Suburb close		-0.120***
		(0.040)
Gothenburg		-0.057
		(0.052)
Skåne		-0.033
		(0.047)
Room		0.070
		(0.048)
Rental property		-0.087***
		(0.033)
Rent		0.011
		(0.011)
Swedish landlord		-0.046
		(0.038)
Landlord unknown ethnicity		-0.042
		(0.054)
No. of days advertised		-0.005***
		(0.002)
Share of foreign borns		-0.005**
		(0.002)
Sent application first		-0.002
		(0.018)
No. of observations	1, 172	1,172
Pseudo R^2^	0.012	0.065

^#^Ali is the reference category

*** = p<0.01,

** = p<0.05,

* = p<0.10

Model 1, where we include only the names of the applicants, shows that Johan had a 16 percentage point higher likelihood than Ali of receiving a callback. Milan and Yong both had an 8 percentage point higher likelihood than Ali. In Model 2, with all control variables, we find that the marginal effects of Johan, Milan, and Yong are stable, i.e., our main results hold also after adding controls. When looking at the overall rental unit characteristics, we find that if a unit was located in the city center or in a suburb close to the city center, the overall likelihood of receiving a callback was 9 and about 12 percentage points lower than if it was located further away. A callback was also less likely if the unit was a rental property. Moreover, the higher the share of foreign-born residents in the municipality/city district where the rental unit was located, the lower the likelihood that an applicant received a callback. Finally, we also controlled for the order in which a landlord received the two applications, and we can conclude that the marginal effect is not significant. We have also run an interaction effect model with the names Johan, Milan, and Yong. The marginal effects of the probit regression with the interactions are shown in Table B2 in S1 Appendix. As can be seen, almost all the interaction terms are non-significant, indicating that, e.g., apartment and landlord characteristics did not affect the difference in likelihood of receiving a callback between Ali and the other applicants.

### 3.3. Pairwise comparisons

Since we submitted two applications for each advertised rental unit, we can also make pairwise comparisons between our applicants’ callback rates. It is not possible for us to be aware of other possible applicants who might have responded to the ads, and this is of course a weakness of the comparisons. However, since we sent out the application pairs on average with about 4-hour intervals, we still believe that it is informative to study whether there are significant differences in response rates between the four applicants depending on in which applicant pair they were placed. Since we have in total four different applicants, we used six different applicant pairs. Table 4 shows the results of the pairwise comparisons within each pair and the p-values from the Wilcoxon matched-pairs signed-rank test.

**Table 4:** Callback rates for each applicant pair. P-values from the Wilcoxon matched-pairs signed-rank test.

Name	Johan vs. Ali	Johan vs. Yong	Johan vs. Milan	Milan vs. Ali	Milan vs. Yong	Ali vs. Yong
Share of callbacks	35% vs. 20%	39% vs. 32%	41% vs. 31%	32% vs. 23%	30% vs. 31%	27% vs. 32%
P-value	0.003	0.189	0.041	0.031	1.000	0.359
No. of obs.	108	99	97	102	104	108

In all combinations in the whole sample, Johan received more callbacks than Ali, Yong, and Milan, and the difference compared with Ali and Milan is significant. When applying for the same rental unit as Milan, Ali has a significantly lower likelihood of receiving a callback. In the other pairs, we do not find any significant differences in callback rates. We conclude that in two of the three possible combinations, Johan has the highest callback rate and Ali the lowest.

To see how the landlords in our study reacted after receiving two application letters close in time, we investigate the shares of landlords who responded to both applicants in a pair (see Table B1 in S1 Appendix). The last row in Table B1 shows that a majority of the landlords answered both housing applicants conditional on having answered at least one of them. The only exception is again Johan and Ali, where only 40 percent of the landlords answered them both. As shown in the Table 3 and in Table B2 in S1 Appendix, we do not have any problem with order effects: The first applicant was not significantly more likely to receive a callback than the second applicant. Finally, we also test by using the test of proportions whether there are significant differences in callback rates *individually* for each applicant depending on in which pair they were included. For example, we are interested in finding out whether Johan received significantly more positive callbacks when paired with Ali than when paired with Milan, or Yong. The results from the two-sample Z proportions test show that this is not the case for any of the applicants. For example, both Yong’s and Milan’s rates of positive callbacks are around 30 percent and Johan’s around 35–40 percent regardless of the name of the other applicant. Thus, there are significant differences in callback rates between some of the pairwise combinations but the rates per applicants are stable across the different combinations. This reinforces our finding that it is the name per se that clearly matters when applying for a rental unit (the results are available on request).

### 3.4. Comparisons over time

Ahmed, Andersson, and Hammarstedt is the study [8] that is closest to ours. In that study, the applicant with an Arab/Muslim-sounding name (Mustafa) received fewer responses from private landlords than the applicant with the exact same application letter but a Swedish-sounding name (Fredrik). Their applicants were, like ours, non-smoking single males without children with a high level of education, a good job, and a good payment history. In both their and our applications, age and the applicant´s work tasks were mentioned. Thus, their applicants sent a similar letter to landlords advertising a rental unit on the same buy-and-sell site (Blocket.se) as we. The main difference between Ahmed, Andersson, and Hammarstedt [8] and our study is that while we sent two application letters for each rental unit, they sent only one application.

Some potentially important changes have happened in Sweden since the study by Ahmed, Andersson, and Hammarstedt [8] was conducted. After the Swedish government in 2013 relaxed the country’s regulation of private landlords, the supply of private rental units increased rapidly [31]. Still, however, the demand for housing has climbed steadily in recent years to a very high level, especially in and close to the largest cities [31]. The share of Swedish municipalities experiencing a deficit in the housing market remained stable at slightly over 40 percent from 2006 to 2013 but then doubled in 2017, at the time of our study. Moreover, the number of immigrants has increased rapidly, especially since 2014 [21].

While the designs of Ahmed, Andersson, and Hammarstedt [8] and our study were very similar, they were not identical. So, we cannot claim that the two studies are directly comparable with each other. We can however conclude that both studies show clear discrimination against Arab/Muslim-sounding names when applying for housing in the Swedish private rental housing market. Moreover, we find clearly lower callback rates for both Johan and Ali (46 and 27 percent, respectively) than for Fredrik and Mustafa in the Ahmed et al. (2010) study (62 and 50 percent, respectively). Thus, both Johan and Ali received fewer callbacks in absolute terms compared with Fredrik and Mustafa. The Ahmed, Andersson, and Hammarstedt study [8] was conducted over 37 days, while our study was conducted over only eight days. This difference might explain the overall lower response rate in our study.

Our aim is to investigate the relative differences in callback rates and whether they have changed over time. When comparing the relative callback rates between Swedish- and Arab/Muslim-sounding names separately, we see that Johan needed to apply for 35 percent more rental units than Fredrik in 2008 to receive a callback. The corresponding number for Ali compared with Mustafa is about 85 percent. By using a z-test, we find that these drops in response rate are significant for both of the two comparisons at the one percent level.

Finally, we believe that one additional difference between the two studies is worth noting: a decrease in the likelihood of receiving a callback over time. In our study conducted in 2017, a male with an Arab/Muslim-sounding name on average needed to respond to 70 percent more rental ads than a male with a Swedish-sounding name before he received a callback, while in 2008, according to the study by Ahmed, Andersson, and Hammarstedt [8] the same figure was 24 percent. While being cautious not to draw too strong conclusions, we claim that the situation for males with Arab/Muslim-sounding names in the Swedish private rental market at least has not improved.

## 4. Conclusions and discussion

Sweden has rapidly changed from being a homogenous country with few non-Nordic natives to having, in the largest cities, up to 33 percent of people having immigrated from a wide range of countries. Moreover, the timing of our study was crucial: The annual number of immigrants who came to Sweden in the years immediately before we carried out our study was higher than ever before or after. In this field experiment, we investigated whether there exists discrimination in the Swedish private rental housing market, and if so, whether there are differences based on the names of rental applicants. We used a correspondent test by randomly sending out equivalent applications from four fictitious, highly educated, and “well-behaved” male applicants to private landlords who had posted for-rent ads on the leading Swedish buy-and-sell website Blocket.se. Each advertiser received an application from two different applicants with names signaling different ethnicities, namely Swedish, Arab/Muslim, Eastern European, and East Asian. The applicants applied for rental units in the three largest metropolitan areas in Sweden. Our results clearly confirm the previous findings that the person with a name associated with the dominant ethnic group received the most callbacks from the private landlords, and especially the Arab/Muslim-sounding name yielded a significantly lower callback rate than the member of the dominant ethnic group. After controlling for the characteristics of the rental units, whether the landlords had a Swedish-sounding name or not, and the region in which the rental unit was located, we found that Johan—a traditional Swedish name—had a 16 percentage point higher likelihood of receiving a callback than Ali. Milan and Yong had a 8 percentage point higher likelihood than Ali.

Moreover, since we sent out two applications to each advertising landlord, we were also able to make pairwise comparisons between our applicants’ callback rates. Our double-application design also made it possible to test whether there were significant differences in callback rates *individually* for each applicant depending on in which pair they were included, but we did not find any such differences for any applicant. Thus, there are several significant differences in callback rates between the fictitious applicants when compared in pairs, but each applicant’s callback rates are stable across the different pair combinations. This reinforces our result that it is the name itself that clearly matters when applying for a rental unit. We conclude that Johan seems to be the most popular among the landlords, followed by both Milan and Yong, despite the fact that all our applicants were equally well-behaved, highly educated persons with good jobs and a steady income. Ali clearly had the lowest rate of positive callbacks and the difference is largest when compared with Johan. Our results confirm the results by Ahmed, Andersson, and Hammarstedt [8] i.e., male applicants with Arab/Muslim-sounding names received fewer callbacks from landlords than persons with a Swedish-sounding name.

Just like in our study, Ahmed, Andersson, and Hammarstedt [8] used the leading Swedish buy-and-sell website Blocket.se and sent similar email letters to private landlords. The designs of our study and their study are however not identical, so we cannot fully compare them. However, we claim that the situation for males with Arab/Muslim-sounding names in the Swedish private rental market has at least not improved in recent years. In our study conducted in 2017, a male with an Arab/Muslim-sounding name on average needed to respond to 70 percent more rental ads than a male with a Swedish-sounding name before he received a callback, while in 2008, the same figure was 24 percent [8].

Numerous effects of some groups being discriminated in the housing market can be noted. Not only does it restrict people’s equal right to housing, it can also lead to an economic cost for both society and individuals. When certain groups face difficulties finding a place to live, they also become restricted in their choice of jobs and life opportunities. If people lack the opportunity to live where they want, they may be forced to also give up job opportunities. In the long run, this can lead to a more segregated society with some groups being left in isolation. Moreover, our results are only valid for well-educated, well-behaved male persons with stable personal finances and do not say anything about the possible discrimination among less skilled persons.

This experiment gave us an opportunity to examine the issue of ethnic discrimination in the private rental housing market. The advantage of the methodology used is that the applicants in the experiment were fictitious, which makes it possible to construct desirable characteristics for the purpose of the study without having to take into account the limitations any real people might have. However, a disadvantage of the methodology is that the results of the experiment do not say anything about who in the end would have been offered a rental contract; instead, all we know is who received a response to his housing application. Moreover, it is not necessarily the case that the information in the submitted applications was enough for a landlord to make a choice based on complete information. Also, as the study is limited to only examining ethnic discrimination among men, more research about discrimination among female applicants in the housing market is needed. In addition, since we sent two applications to each landlord, there was a risk that landlords could become suspicious, which in turn could have affected the results. While we cannot fully exclude this possible problem, our results show that a majority of the landlords actually answered both applicants in each pair combination, and that the order in which the applicants submitted their letters did not affect the results. As for our two objectives, we find heterogeneity in discrimination in the housing market across the different immigrant groups and males with Arabic/Muslim-sounding names have the most difficulties receiving callbacks in the Swedish private rental market. Secondly, the comparisons with the Ahmed et al. (2010) paper shows that the situation for a male person with an Arabic/Muslim-sounding name has at least not improved in Sweden in the past decade. More likely, it has instead become more difficult to rent an apartment in the private housing market.

## Supporting information

S1 Appendix (DOCX)

S1 File (DO)

S2 File (DO)

S3 File (DTA)

S4 File (DTA)
