# Supplementary material for: Name matters! The cost of having a foreign-sounding name in the Swedish private housing market
Source: PLoS One. 2022 Jun 8;17(6):e0268840. doi: 10.1371/journal.pone.0268840 (PMC9176839; doi:10.1371/journal.pone.0268840)
Supplement: S1 Appendix — (DOCX) [file pone.0268840.s001.docx]

# Appendix A

**Application email 1:**

Hi!

My name is Johan/Ali/Milan/Yong and I’m very interested in the apartment you have for rent. I hold a permanent position as a business economist at the SEB bank, and because I’m just about to transfer to the company’s office in Stockholm/Gothenburg/Malmö, and I’m looking for housing in the area. I’m a 27-year-old man with a business degree, a steady income, and excellent credit. In my spare time I enjoy working out and spending time with friends. I’m single, a non-smoker, and I don’t have any pets or children. As a person, I’m easygoing and like to keep things around me neat and tidy. As for a possible move-in date, I’m quite flexible as I really want to rent your apartment.

I’m looking forward to hearing from you!

**Application email 2:**

Hi!

My name is Johan/Ali/Milan/Yong and I’m extremely interested in renting the apartment you have advertised on Blocket.se! I currently live alone and in my spare time I enjoy working out and spending time with friends. Other than that, I’m pretty calm and laidback. I don’t smoke, I don’t have any pets or children, and I like to keep things clean and well organized. I’m a 27-year-old male with a university degree in business administration, a steady income (I currently hold a permanent position), and very good credit. The reason I’m looking for housing in your area is that I have been offered a position as an accountant at KPMG. I’m quite flexible and confident we will be able to agree on a move-in date that will work for both of us.

Please contact me so we can talk more!

Johan/Ali/Milan/Yong

**Application email 3:**

Hi.

I’m looking for a place to stay and am very interested in the apartment you have for rent. I believe it will fit me perfectly. My name is Johan/Ali/Milan/Yong and I’m a 27-year-old guy who has just been hired as an adviser at Söderberg & Partners, which is the reason I’m looking for a home. I have a degree in business and have a steady and reliable income. My credit is excellent. I am a person who likes to keep things in good order and I never miss any payments. In my off-time, I like to exercise and hang out with friends and family. I don’t smoke and am currently not in a relationship. I also do not have any pets or children. When it comes to a good move-in date, I’m pretty flexible and open to suggestions.

Please contact me so I can tell you more about myself.

Application email used in the Ahmed et al. (2010) study:

Hi,

My name is Mustafa Karim (Fredrik Karlsson) and I am 35 years old. I would like to sign up as interested in renting the advertised apartment. I am an economics graduate and I have been working as an advisor at a bank for eight years. I am single, no children, nonsmoking, and no payment complaints. Good references are available.

Sincerely,

Mustafa Karim (Fredrik Karlsson).

# Appendix B

Table B1. Callback rates for both applicants in each pair combination, in the whole sample (unconditional) and conditional on a landlord giving a callback.

| Name | Johan vs. Ali | Johan vs. Yong | Johan vs. Milan | Milan vs. Ali | Milan vs. Yong | Ali vs. Yong |
| --- | --- | --- | --- | --- | --- | --- |
| Share of callbacks for both applicants, whole sample | 16% | 25% | 26% | 19% | 22% | 20% |
|  |  |  |  |  |  |  |
| No. of obs. | 108 | 99 | 97 | 102 | 104 | 108 |
| Share of callbacks for both applicants, conditional on at least one callback | 40% | 54% | 54% | 51% | 58% | 52% |
| No. of obs. | 43 | 42 | 46 | 37 | 40 | 42 |

| Table B2. Marginal effects after probit regression with interaction terms. Standard deviations in parentheses. Standard deviations clustered on advertisement level. | |
| --- | --- |
| Variable | Marginal effect  (Std.dev.) |
| Johan# | 0.103  (0.251) |
| Milan# | -0.148  (0.272) |
| Yong# | -0.392  (0.246) |
| Inner city | -0.028  (0.080) |
| Inner city *Johan | -0.121  (0.108) |
| Inner city * Milan | -0.067  (0.096) |
| Inner city * Yong | -0.047  (0.106) |
| Suburb close | -0.045  (0.064) |
| Suburb close * Johan | -0.104  (0.084) |
| Suburb close* Milan | -0.157*  (0.081) |
| Suburb close* Yong | -0.009  (0.077) |
| Gothenburg | -0.040  (0.084) |
| Gothenburg* Johan | 0.040  (0.104) |
| Gothenburg* Milan | -0.110  (0.105) |
| Gothenburg* Yong | 0.006  (0.103) |
| Skåne | -0.005  (0.081) |
| Skåne * Johan | -0.015  (0.105) |
| Skåne* Milan | -0.109  (0.102) |
| Skåne * Yong | 0.024  (0.099) |
| Room | -0.016  (0.077) |
| Room *Johan | 0.097  (0.097) |
| Room*Milan | 0.135  (0.094) |
| Room*Yong | 0.113  (0.097) |
| Rental property | -0.137**  (0.056) |
| Rental property*Johan | 0.023  (0.072) |
| Rental property*Milan | 0.047  (0.071) |
| Rental property*Yong | 0.111  (0.069) |
| Rent | -0.022  (0.018) |
| Rent*Johan | 0.034  (0.023) |
| Rent*Milan | 0.035  (0.022) |
| Rent*Yong | 0.060**  (0.023) |
| Swedish landlord | 0.003  (0.061) |
| Swedish landlord*Johan | -0.005  (0.081) |
| Swedish landlord*Milan | -0.082  (0.078) |
| Swedish landlord*Yong | -0.097  (0.076) |
| Landlord unknown ethnicity | 0.068  (0.086) |
| Landlord unknown ethnicity*Johan | -0.159  (0.113) |
| Landlord unknown ethnicity*Milan | -0.117  (0.115) |
| Landlord unknown ethnicity*Yong | -0.165  (0.110) |
| No. of days advertised | -0.004*  (0.002) |
| No. of days advertised*Johan | -0.001  (0.003) |
| No. of days advertised*Milan | -0.001  (0.003) |
| No. of days advertised*Yong | -0.0001  (0.003) |
| Share of foreign born | -0.005  (0.004) |
| Share of foreign born*Johan | -0.005  (0.005) |
| Share of foreign born*Milan | -0.004  (0.005) |
| Share of foreign born*Yong | -0.002  (0.005) |
| Sent application first | 0.014  (0.054) |
| Sent application first * Johan | -0.017  (0.079) |
| Sent application first * Milan | -0.010  (0.083) |
| Sent application first * Yong | -0.020  (0.079) |
| Number of obs. | 1,172 |
| Pseudo R2 | 0.084 |
| ***= p<0.01, **=p<0.05, *= p<0.10  #Ali is the reference category | |
